# Heavy Metal Contamination in Leafy Vegetables Grown in Jazan Region of Saudi Arabia: Assessment of Possible Human Health Hazards

**DOI:** 10.3390/ijerph20042984

**Published:** 2023-02-08

**Authors:** Asim Najmi, Mohammed Albratty, Abdul Jabbar Al-Rajab, Hassan A. Alhazmi, Sadique A. Javed, Waquar Ahsan, Zia ur Rehman, Rym Hassani, Saad S. Alqahtani

**Affiliations:** 1 Department of Pharmaceutical Chemistry and Pharmacognosy, Faculty of Pharmacy, Jazan University, Jazan 45142, Saudi Arabia; 2 Centre for Environmental Research and Studies, Jazan University, Jazan 45142, Saudi Arabia; 3 Etcetera Publications, Chesterville, ON K0C1H0, Canada; 4 Substance Abuse and Toxicology Research Center, Jazan University, Jazan 45142, Saudi Arabia; 5 Nursing Department, University College of Sabya, Jazan University, Jazan 45142, Saudi Arabia; 6 Department of Pharmacy Practice, College of Pharmacy, Jazan University, Jazan 45142, Saudi Arabia; 7 Pharmacy Practice Research Unit, College of Pharmacy, Jazan University, Jazan 45142, Saudi Arabia

**Keywords:** heavy metals, ICP-MS, Jazan, leafy vegetables, Saudi Arabia, target hazard quotient

## Abstract

The food chain, through vegetable consumption, is considered to be an important route of heavy metal exposure. Therefore, in this study, heavy metal concentrations in leafy vegetables grown in the Jazan region of Saudi Arabia were assessed using an ICP-MS. Lettuce, radish, mint, parsley and jarjir (Arugula) were selected for study and subjected to digestion using HCl. The results indicated that the Fe level was highest in all vegetables, while jarjir was the most contaminated vegetable. However, no tested metal exceeded the maximum permissible limits set by the FAO/WHO and European Committee. The possible health hazards associated with the exposure to metal contaminants via vegetable consumption were evaluated by estimating target hazard quotient (THQ) values, and the results revealed that the vegetables grown in close proximity of Jazan city were the most contaminated and those in Darb the least. However, the daily intakes of all the tested metals were well below the corresponding oral reference doses (RfDs), and the THQ values were less than unity, suggesting that the vegetables grown in the studied region were safe and the heavy metal exposure via vegetable consumption was unlikely to cause adverse effects to the local inhabitants of the region.

## 1. Introduction

The health hazards due to heavy metal exposure are well known, and their intake through the ingestion of foodstuffs is reported throughout the world [1,2,3]. Being persistent and non-biodegradable in nature, heavy metals keep accumulating in biological systems, affecting their normal physiology. Their accumulation in the human body beyond certain limits adversely affects vital organs, such as the liver, brain and kidneys, bone and blood. Excessive intake of certain heavy metals including cadmium, lead, copper and chromium can cause serious adverse effects including bone, neurological and cardiovascular disorders, renal impairment and gastrointestinal disturbances. Depending on the exposure time, the toxicity due to heavy metal exposure may be acute or chronic [4,5,6]. Certain studies on metal toxicity have revealed that heavy metals are neurotoxic, mutagenic, teratogenic and carcinogenic [7]. Because of their role in the biological systems, Zn, Cu and Mn are considered to be essential elements; however, their accumulation in excess amounts in the body may produce serious toxicity in humans and animals [8]. Excessive exposure of Cd can adversely affect the liver and lungs and produce nephrotoxicity, immune disorder and bone toxicity [9,10]. The long-term exposure of lead can cause cardiovascular complications and nephropathy and affect the intellectual growth of children [11,12,13]. Higher Zn exposure can adversely affect the immune system and reduces the level of HDL in the body, and an elevated Cu concentration in the body can induce liver damage and gastrointestinal disturbances [14,15].

Vegetables are essential components of the human diet due to their high nutritional value, dietary fibers and antioxidant contents. Characteristically, trace elements along with essential nutrients are absorbed by plants; hence, the quality and safety of vegetables is of great concern because of trace element contamination from soil and atmospheric deposition [16]. The concentration of heavy metals in the soil, type of soil, atmospheric depositions, climate, plant species and stage of plant maturity at harvesting time are the main factors affecting the bioaccumulation of heavy metals in vegetables [17,18,19]. Additionally, sewage sludge and the application of fertilizers, pesticides and manure may also influence the intake of trace elements by the plants as they modify the soil properties such as organic matter and pH as well as affect the concentration of metals present in the soil. It was found that the uptake of metals by crops is directly linked to the heavy metal concentration in the soil [20,21]. Furthermore, the exposure of post-harvest vegetables to air pollution during transportation and sales may also result in increased metal contamination [22]. In a study conducted in the city of Riyadh, Saudi Arabia, it was reported that the unwashed leafy vegetable from roadside markets had a higher concentration of heavy metals than washed vegetables [23]. The metal contaminants suspended in the environment can be deposited on the top soil which may be taken up by the plants or alternatively get deposited on the leaves and other aerial parts of the plant and get absorbed. Larsen et al. (1992) [24] and Sanchez-Camazano et al. (1994) [25] have reported a positive correlation between atmospheric metal deposition and elevated levels of heavy metals in soil and plants. A higher accumulation of cadmium, lead and chromium as a result of atmospheric deposition in leafy vegetables was reported by Voutsa et al. (1996) [18]. In general, leafy vegetables accumulate higher concentrations of contaminant elements than fruits, seeds and grains [26,27]. The enhanced atmospheric deposition of contaminants in the soil and hence increased metal concentrations in the edible parts of vegetables is directly correlated to increased human activities.

However, a number of investigations have been reported assessing heavy metal contamination in vegetables cultivated across the globe [2,8,23,25,26,28,29,30]; to our knowledge, no study on leafy vegetables has been carried out so far in the Jazan region of Saudi Arabia. The leafy vegetables constitute an important foodstuff, mainly consumed in the form of salad by the local population of the tested region. Therefore, in order to ensure food safety, it is extremely important to monitor the heavy metal contamination in vegetables along with their associated health risks for the consumers. Furthermore, the studied region is the extreme southwest corner of Saudi Arabia with a considerable amount of land lying at the coast of the Red Sea. It has relatively lower infrastructure development in comparison to other parts of the country. However, since the last few years the region has been experiencing speedy development following the declaration of the economic zone in the region. A number of industries have been set up in the region leading to increased human activities. The Jazan region is rich in vegetation, and the cultivation of a variety of vegetables, fruits and food grains is a common practice. Although it is not considered a polluted region, there is a lack of data related to heavy metal pollution. Due to speedy industrial and infrastructural development in the region, it is thought that the plants grown in the region might get contaminated from heavy metals. As it is a well-known fact that even chronic low-level intakes of heavy metals might have damaging effects on human health, regular monitoring of these toxic elements is necessary in food items including vegetables. Therefore, this study was carried out to measure the levels of Cd, Co, Cr, Cu, Mg, Fe, Mn, Pb, Ni and Zn contamination in the edible portion of leafy vegetables cultivated from across the Jazan region and to estimate their contribution to the daily intake of the metal. The potential health risks posed by these elements for the local inhabitants via leafy vegetable intake were assessed, and the pollution load of the vegetables was estimated by calculating the metal pollution index (MPI). The acquired data from this investigation may be helpful in providing important information to environmental agencies to update their policies and programs for assured food safety and quality as well as to enhance awareness among the community of the hazards of environmental pollution.

## 2. Materials and Methods

### 2.1. Chemicals and Instruments

All solutions and dilutions used in the experiment were prepared in deionized ultra-pure water produced in-house using Milli-Q water purification system (Millipore, USA). The chemicals and reagents used were of analytical grade (AR) and used as such without further purification. The conc. HCl used for digestion of the samples was procured from Sigma-Aldrich, Germany. The reference standards (1000 mg/L in nitric acid) for Cd, Pb, Co, Cr, Cu, Fe, Mn, Mg and Zn were purchased from Sigma-Aldrich, Schnelldorf, Germany. Before their use in the experiment, all glassware and plasticware were treated with acid by soaking in 10% HNO_3_ overnight then washed thoroughly using deionized water and dried. The concentrations of tested metals in vegetable samples were measured using an inductively coupled plasma mass spectrometer (ICP-MS, model number: 7500a, Agilent Technologies Inc., Waldbronn, Germany). The instrument was monitored with ChemStation Software (Agilent Technologies Inc., Waldbronn, Germany).

### 2.2. Study Area

**Figure 1:**
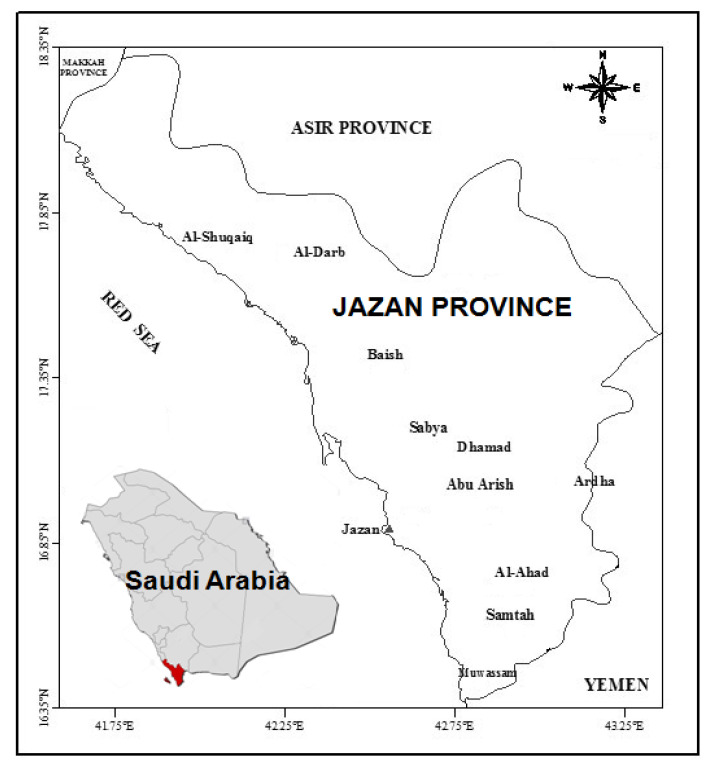
The map displaying Jazan province situated along the coast of the Red Sea in the southern region of Saudi Arabia.

The present study was conducted in the Jazan region which is one of the thirteen provinces of Saudi Arabia. Jazan city is the capital of the province which is situated in the southwest region (16°53′ N and 42°33′ E) of the country. The province is directly connected to the northern border of Yemen and lies along the coast of the Red Sea (Figure 1). The climate is arid tropical, and the mean annual temperature of the region is 30.1 °C. Jazan is the economic city of the region and experiencing increased industrial development and commercial activities over the last few years. The region is one of the major agricultural producers of the country, and a variety of food grains, fruits and vegetables are cultivated. In addition to that, the city has copper, aluminum and steel industries, along with a big power station, seaport, oil port, desalination plant and massive automobile factories [31,32].

### 2.3. Sample Collection

Twenty-six samples (approximately 500 g each) of edible portions of leafy vegetables including lettuce (*Lactuca sativa*), mint (*Mentha piperita*), jarjir (Arugula, *Eruca sativa*), radish (*Raphanus sativus*) and parsley (*Petroselinum crispum*) were collected from five locations (Jazan, Abuarish, Sabya, Damad and Darb) across the Jazan province of Saudi Arabia. The vegetable samples were collected in the months of April and May 2022 and kept in clean polyethylene bags until further use. From each sampling location, 3–6 cultivation plots were chosen, and approximately 100–150 g of each vegetable was collected from four different sites of every plot, and composite samples were made by mixing thoroughly. The number of composite samples collected from each location were as follows: Jazan, *n* = 4; Sabya, *n* = 5; Abuarish, *n* = 6; Damad, *n* = 5; and Darb, *n* = 6. The collected samples were directly brought to the laboratory, and the adhered soil and airborne dust were cleaned by washing first with running tap water and then with deionized water. The collected vegetables were chopped into small pieces, dried in an oven at 60 °C until a constant weight, crushed to pass through a 40-mesh sieve and stored in a refrigerator for further processing.

### 2.4. Method Validation

The ICP-MS method applied in this investigation was validated as per ICH Q2 (R1) guidelines [33] for sensitivity, linearity, precision and accuracy. Calibration standard solutions in the concentration range of 5–100 ppb (7 points) for each trace element were prepared using the respective reference standards to determine the linearity, and all the solutions were analyzed with an ICP-MS. A calibration curve for each metal was constructed by plotting detector signal against corresponding concentration, and linear regression analysis was used to obtain the correlation coefficient (R^2^). The detection limit (LOD) of the method was estimated using the standard deviation and slope of the calibration line. The quality control solutions of concentrations 5, 20, 60, 90 ppb for each metal ion were injected in triplicate to assess the precision and accuracy of the method. The percent RSD of the repeated analysis was considered as precision, whereas the method accuracy was estimated by using Equation (1).

(1) $$\%\;Accuracy=\frac{Practiccally\;observed\;concentration}{Nominal\;concentration\;}\times 100$$

For each metal, a recovery experiment was conducted by adding fixed quantities of metal standards in the vegetable samples. After sample digestion and treatment as mentioned above, the spiked and non-spiked samples were injected into the system for analysis by using the developed method. The % recovery of each metal was determined by using Equation (2).

(2) $$\%\;Recovery=\frac{Conc.\;in\;spiked\;sample-Conc.\;in\;nonspiked\;sample\;}{Amoubt\;added\;}\times 100\;\;\;\;\;\;\;\;\;\;\;$$

### 2.5. Preparation of Samples and Determination of Heavy Metal Contents

Accurately weighed 1 g of powdered leafy vegetable sample was transferred to a silica crucible, placed in a muffle furnace and ashed for 12 h at 450 °C. The crucible was taken out and allowed to cool and 5 mL of hydrochloric acid (20%) was added and left overnight at laboratory temperature (25 ± 2 °C) for digestion. The digested content was filtered into a volumetric flask (50 mL), and the total volume was adjusted up to the mark using deionized water [34]. The procedure was repeated to prepare all vegetable samples and the blank (omitting the sample). The following instrumental conditions were set prior to analysis: carrier gas—argon; RF power—1300 W; spray chamber type—concentric; auxiliary gas flow—1 L/min; nebulizer gas flow—0.6 L/min; plasma gas flow—15 L/min; wavelength range—166-847 nm; plasma viewing—duo (axial/radial); read delay—30 s; and integration time—maximum 5 s. All the prepared solutions were analyzed by the developed method to determine the concentrations of nine trace elements, Cd, Co, Cr, Cu, Mg, Fe, Mn, Pb and Zn using an ICP-MS. Each sample was analyzed in triplicate, appropriate dilution factors were applied in the calculation of results, and the final concentration of each element was considered as the mean of three measurements. The concentrations of tested elements in this study were reported in mg/kg on a dry weight (DW) basis, unless mentioned. The respective wavelengths at which the selected elements were measured are given in Table 1.

### 2.6. Statistical Analyses

Mean, minimum and maximum heavy metal concentrations were calculated using Microsoft Excel 2010 program. One-way analysis of variance (ANOVA) was used to evaluate the significant variation in heavy metal concentrations in samples from different locations. Pearson test was performed to establish the correlation between heavy metal concentrations in different leafy vegetables, and the correlation coefficient (r) was estimated using Microsoft Excel 2010 program. The concentrations of heavy metals present in the leafy vegetables from different locations in the Jazan region were further analyzed by a widely used multivariate chemometric method using NCSS statistical program v. 2022 (NCSS LLC, Kaysville, UT, USA). Hierarchical cluster analysis (HCA) was performed on the obtained data which divided the variables into different clusters on the basis of their characteristics. In this analysis, five types of vegetables from five different locations constituted twenty-five columns, whereas the concentration of metals constituted the eight rows. A dendrogram plot was constructed using average linkage between the variables and their agglomeration schedule in order to measure the cohesiveness of the formed clusters. The unweighted pair-group (group average) method was used as the clustering method with Euclidean as distance type and standard deviation as scale type.

### 2.7. Metal Pollution Index (MPI)

MPI is a parameter to assess the overall load of heavy metals in vegetable samples. The MPI value for each vegetable was calculated using the following mathematical Equation (3) [29]:

(3) MPImgkg=CCd×CCo×CCr×CCu×CFe×CMn×CPb×CZn1n                        

where *C_Cd_*, *C_Co_*, *C_Cr_*, *C_Cu_*, *C_Fe_*, *C_Mn_*, *C_Pb_* and *C_Zn_* are the average concentrations of Cd, Co, Cr, Cu, Fe, Mn, Pb and Zn, respectively, in vegetable samples from all the tested locations, and *‘n’* is the number of metals included in the assessment (*n* = 8).

### 2.8. Potential Health Risk Assessment

An assessment of health risk estimates the possible adverse effects on human health as a consequence of exposure to pollution hazards [35]. In the health risk assessment, the contaminants were classified as non-carcinogenic and carcinogenic. In the present investigation, the non-carcinogenic hazards due to heavy metals were assessed on the basis of daily exposure to these elements through intake of leafy vegetables grown in the Jazan area. The assessment was executed according to the standards set by the United States Environmental Protection Agency (US EPA) [36]. The estimated daily intake (EDI) of heavy metals through vegetable consumption depends on the metal content and the amount of the vegetable consumed on a daily basis. The EDI (mg/kg/day) of tested heavy metals was calculated using the following Equation (4):

(4) $$Estimated\;Daily\;Intake\;\left(EDI\right)=\frac{C\times C_{f}\times IngR}{BW}\;\;\;\;\;\;\;\;\;\;\;\;\;\;\;\;\;\;\;\;\;\;\;\;\;\;$$

where *C* (mg/kg, dry weight basis): average metal concentration in the tested leafy vegetables; *C_f_* (0.085): conversion factor used to convert the fresh to dry weight of the tested leafy vegetables [37]; *IngR* (kg/person/day): daily average intake of the vegetables; and *BW*: average body weight of the target population, for adults, 70 kg [36] and for children, 32.7 kg [38]. The average daily consumption of vegetables was considered as 0.20 kg/person/day for adults [37], whereas the daily intake rate for children was taken as 0.133 kg/person/day.

The non-carcinogenic risks to human health via the consumption of metal-contaminated vegetables were estimated on the basis of the hazard quotient (HQ), which is defined as the health risk due to exposure of a pollutant with respect to the estimated daily intake (EDI). If the hazard quotient of a particular contaminant is less than one (<1), there would be no obvious adverse effects expected on the exposed population. The HQ was calculated by dividing the estimated daily intake (mg/kg/day) of the contaminant through vegetable ingestion by the reference oral dose, as follows (Equation (5)):

(5) $$HQ=\frac{EDI}{RfD}\;\;\;\;\;\;\;\;\;\;\;\;\;\;\;\;\;\;\;\;\;\;\;\;\;\;\;\;\;\;\;\;\;\;\;\;\;\;\;\;\;\;\;\;\;\;\;\;\;\;\;\;$$

The reference oral dose (*RfD*, mg/kg/day) is an estimated value of tolerable daily ingestion of pollutants (maximum permissible risk) by human beings during a lifetime. The values of *RfD* used in this study were taken from De Miguel et al. (2007) [39] (for metals Cd, Cr, Cu, Mn, Pb and Zn) and Sharma et al. (2016) [29] (for metals Fe and Co). 

## 3. Results and Discussion

### 3.1. Method Validation

The parameters including sensitivity, linearity, precision and accuracy were evaluated by following the ICH tripartite guidelines Q2 (R1). The developed method exhibited good sensitivity as the detection limit of all the tested metals was calculated to be below 0.28 ppb. This reflected the suitability of the method for determining the concentration of these trace elements in vegetable samples. The calibration curves prepared for each heavy metal exhibited excellent linearity and a correlation coefficient R^2^ of ˃ 0.99 was obtained. The percent RSD values of the repeated analysis of quality control samples were between 0.42 and 1.68%, and the accuracy was in the range of 95.9–109.3%. The percent recoveries for all the metals were in the range of 92.6–107.4%. These values indicated acceptable precision and accuracy of the method.

### 3.2. Heavy Metal Concentrations

Atmospheric deposition is considered the major source of metal contamination in agricultural soils and plants near urban locations and industrial areas. The levels of heavy metals in the agricultural soil and hence in the crop are also influenced by the type of water used for irrigation. The literature survey revealed that the vegetables irrigated with sewage water contained higher heavy metal contents than those irrigated with clean tube-well water. Furthermore, the difference in the heavy metal contents in the different vegetables could also be explained by the varying capacity of the plants to absorb and accumulate metals and due to variation in their growth rates causing different plant species to behave differently in accumulating environmental contaminants in their tissues [40,41]. Even different plant parts have different degrees of uptake, translocation and accumulation of heavy metals, and the magnitudes of these processes vary from one species to another. Additionally, soil properties such as pH and the organic matter content affect the adsorption and accumulation of heavy metals in soil and so in the plant parts [42]. As far as the uptake of contaminants by plant is concerned, the plants are divided into two groups including ‘accumulators’ and ‘excluders’. The former category tolerate the accumulation of contaminants in their aerial parts and biodegrade the contaminants into an inactive form; the latter ones survive by limiting the uptake of the contaminants into their biomass [43]. Heavy metals such as Pb, Cd, Co, Ni, Mn and Zn can be accumulated at 100 to 1000 times higher concentrations by metal accumulator plant species than by excluders. The microorganisms present in the rhizosphere might help to mobilize these metal ions and increase their bioavailability [44,45]. Plants have efficient and specific mechanisms for the uptake of important micronutrients from the surrounding soil, even when existing at very low levels.

Chelating agents produced by plants, changes the pH and redox reactions occurring in the roots might help to dissolve and adsorb the micronutrients from the surrounding soil. Plants also have efficient mechanisms for the translocation and storage of micronutrients in different parts of the plant. The same mechanism also contributes to the uptake, translocation and storage of toxic contaminants including heavy metals [46]. The long-term application of pesticides and fertilizers affect the concentration of toxic elements in the agricultural soil [47,48]. Previous studies reported that higher accumulation of Zn, Cu, As and Pb in agricultural top soil was due to excessive application of herbicides and pesticides [49]. Another study reported that the concentration of Cr was greater in paddy roots in comparison to the shoot part including the stem and the part 10 cm above the root. This was due to the redox processes in the plant which led to the translocation of metals from the root to the shoot part [50]. Furthermore, another study reported that the reaction of Cr with the –COOH group in plants can disrupt the movement of Cr from root to shoot, resulting in a lower accumulation of Cr in the shoot part [51]. Most of the metals were found to accumulate more in the roots of the paddy plant, except Zn which was found to accumulate in the stem at greater concentrations [52].

Jazan province is among the agriculturally rich regions of Saudi Arabia, in which a wide variety of vegetables, fruits and food grains are cultivated. Throughout the country, there is a strong trend of eating non-vegetarian food; hence, leafy vegetables are consumed as a green salad by the inhabitants as they provide essential fibers. In the current study, contamination levels of nine metals in the edible parts of leafy vegetables including lettuce, radish, mint, parsley and jarjir cultivated from the region were measured, and the observed concentration range of the metals is summarized in Table 2. The samples were prepared by the digestion technique using HCl and analyzed using an ICP-MS. The average contents of the tested heavy metals in the leafy vegetables in this study ranged widely, with values of 0.242–1.249 mg/kg for Cd, 0.457–0.764 mg/kg for Co, 1.10–3.534 mg/kg for Cr, 5.36–9.18 mg/kg for Cu, 355.4–1195.9 mg/kg for Fe, 31.90–132.63 mg/kg for Mn, 0.858–1.175 mg/kg for Pb and 18.20–36.72 mg/kg for Zn (on a dry weight basis). Mg was recorded below the detection limit. The overall concentrations of the metals were in the order of iron > manganese > zinc > copper > chromium > lead > cobalt > cadmium > magnesium. Leafy vegetables were selected for this study as they tend to accumulate higher concentrations of trace metals than other vegetable types. Previous studies indicated that leafy vegetables can be used as bioindicators of soil pollution, and such investigations must be prioritized to draw attention to public health risks [53]. This is due to the fact that the transfer of metal contaminants from root to stem and then to fruits and flower parts takes a longer time which results in low accumulations; however, leafy vegetables have comparatively greater translocation and transportation rates than other vegetables [54].

**Figure 2:**
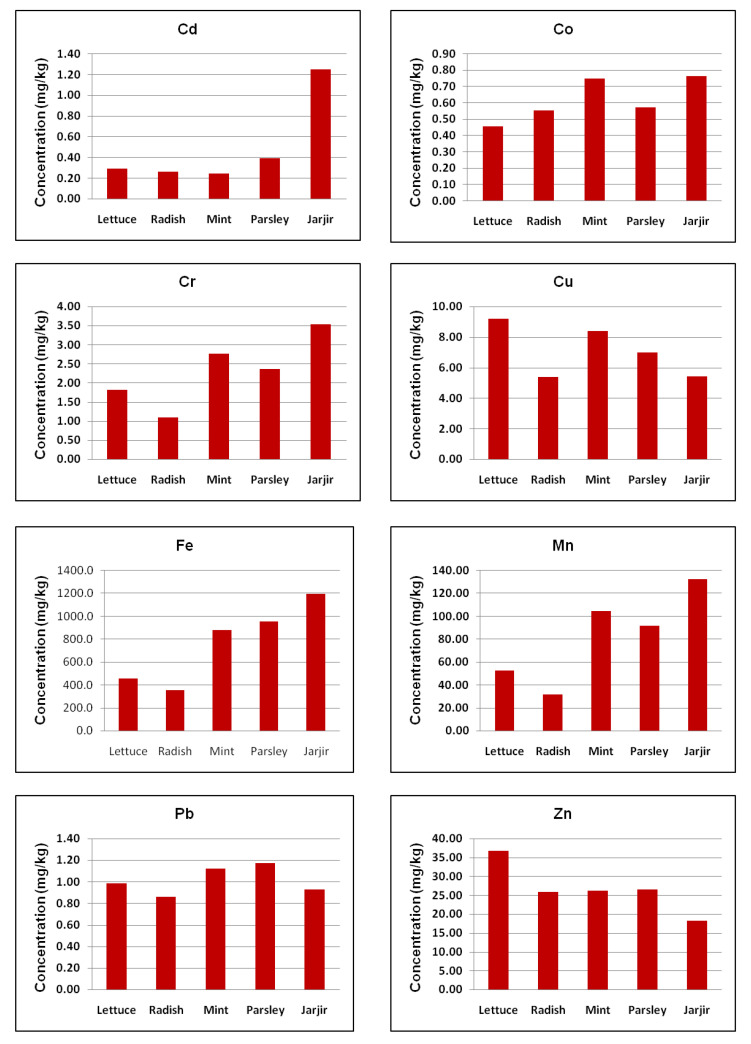
Graph representing the heavy metal distribution in leafy vegetables; the mean concentrations (on dry weight basis) include all the tested sites.

Cu, Fe, Mn and Zn are considered to be essential micronutrients for all living organisms. Accordingly, compared with other metals, these elements were observed in relatively higher concentrations in the vegetable samples. As shown in Figure 2, Fe was the most abundant metal detected in different leafy vegetables, with the highest mean concentration of 1195.88 mg/kg observed in jarjir and the lowest mean level of 355.38 mg/kg present in radish. The lowest and highest average Mn concentrations were measured as 31.90 mg/kg and 132.63 mg/kg in radish and jarjir, respectively, while the minimum and maximum Cu concentrations were 5.36 mg/kg and 9.18 mg/kg in radish and lettuce, respectively. The minimum and maximum contents of Zn were 18.20 mg/kg and 36.72 mg/kg in jarjir and lettuce, respectively. Pb and Cd are considered to be potentially toxic metals because of their persistence, high toxicity and bioavailability [55,56]. In the tested vegetable samples, the highest and lowest average concentrations of Cd were found in jarjir (1.25 mg/kg) and mint (0.24 mg/kg), while the highest Pb concentration (1.18 mg/kg) was detected in parsley. Radish was found to be the least contaminated by Pb, with a mean concentration of 0.86 mg/kg. The observed lowest and highest Co levels were 0.46 mg/kg and 0.76 mg/kg in lettuce and jarjir, respectively, while the maximum and minimum concentrations of Cr were measured to be 3.53 mg/kg and 1.10 mg/kg in jarjir and radish, respectively. Additionally, jarjir was found to be the most contaminated with heavy metals altogether, whereas radish was the least. On the basis of the concentrations of the heavy metals observed in the present investigation, the leafy vegetables could be arranged in the following order: jarjir > parsley > mint > lettuce > radish. Jarjir is one of the most popular leafy vegetables, regularly consumed as an ingredient of green salad by the local inhabitants.

### 3.3. Geographical Distribution of Metal Contents

**Figure 3:**
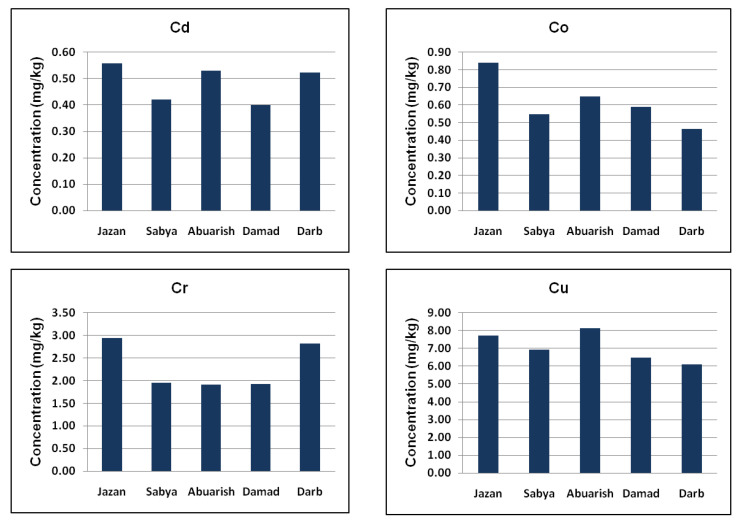

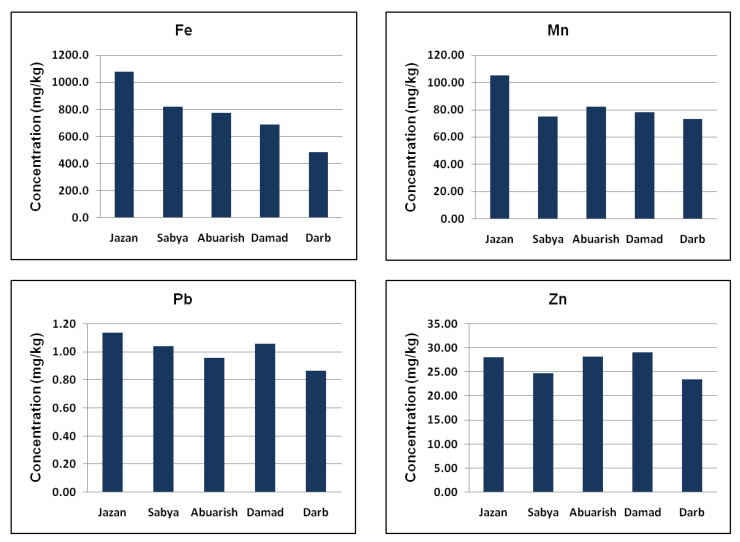
Location-based distribution of heavy metals within the studied region; mean concentrations (on dry weight basis) measured in leafy vegetables including lettuce, radish, mint, parsley and jarjir.

To investigate the geographical distribution of the tested trace elements, Jazan region was divided into five cities, namely Jazan, Sabya, Abuarish, Damad and Darb. As shown in Figure 3, the highest average concentrations of Cd, Co, Cr, Fe, Mn and Pb were recorded in the vegetable samples collected from locations close to Jazan city. The concentration of Cu was found to be highest in the Abuarish region, while the most contaminated region with Zn was Damad. In general, the vegetable samples from Darb were found to be the least contaminated, while the highest levels of the heavy metals were measured in the samples collected in close proximity to Jazan city. Altogether, on the basis of the heavy metal concentrations, the studied locations can be arranged in the following order: Jazan > Sabya > Abuarish > Damad > Darb. The possible reasons for the elevated heavy metal levels nearby Jazan city include its being the capital of the province and experiencing higher industrial and economic growth than other sites of the studied region. Apart from that, the city has a higher population density, road traffic, vigorous construction activities and automobile factories, in addition to a huge power house, gasoline station, seaport and some metal-based industries. 

The heavy metal concentrations measured in this study were compared with their recommended highest permissible intake set by the European Committee (EC) [57] and Food and Agriculture Organization (FAO/WHO) [58]. The observed metal levels in the tested samples were found to be considerably lower than the maximum permissible limits. This suggested that all the tested vegetables were unpolluted and the metals present were within the safe limit. The EC permits maximum concentration values for Cd, Cu, Pb and Zn of 0.2, 20, 0.3 and 50 mg/kg (on a fresh weight basis), respectively, while the FAO/WHO limits for Cd, Cr, Cu, Pb and Zn are 0.2, 2.3, 9.4, 0.3 and 73.3 mg/kg (on a fresh weight basis), respectively. In comparison to these values, the average levels of Cd, Co, Cr, Cu, Fe, Mg, Mn, Pb and Zn in the edible parts of leafy vegetables recorded in this study were 0.041, 0.053, 0.197, 0.60, 65.4, BDL, 7.02, 0.086 and 2.271 mg/kg (on a fresh weight basis), respectively. The conversion factor of fresh to dry weight was taken as 0.085 [37]. The recommended limits for Fe, Mg and Mn were not found; however, Fe and Mn are among the most abundant metals naturally found in the earth’s crust. 

The heavy metal concentrations in the edible portions of the tested vegetables measured in this study were also compared with some previous reports from across the world. The levels of tested heavy metals in the present investigation were found to be lower than those reported in green vegetables in previous studies, except the levels of Fe and Mn which were found to be lower in a few studies (Table 3). For example, the concentrations of Cd, Cu, Pd and Zn in green vegetables grown in Dar es Salaam, Tanzania ranged from 0.1 to 0.6, 2.5 to 16, 1.9 to 6.6 and 14.8 to 49.3 mg/kg on a dry weight basis [34]. Ali and Al-Qahtani (2012) reported higher results (0.93 to 4.13 mg/kg for Cd, 2.06 to 33.22 mg/kg for Cu, 0.54 to 6.98 mg/kg for Pb and 8.27 to 71.77 mg/kg for Zn) in green vegetables collected from different regions of Saudi Arabian markets, while in the same study lower concentrations of Fe (31.96 to 543.5 mg/kg) and Mn (4.16 to 94.16 mg/kg) were reported [59]. Higher concentrations of Cd, Cr, Cu, Pb and Zn were observed in leafy vegetables collected from the city of Harare, Zimbabwe [60]. Similarly, Rattan et al. (2005) [37] reported considerably lower concentrations of Fe and Mn in vegetables collected from Delhi, India, whereas significantly higher levels of Cu and Zn were observed. A lower Cr content (BDL −0.48 mg/kg, fresh weight basis) than that observed in this study was reported in the vegetable samples from Sao Paulo State, Brazil, while Cd, Co and Pd were found to be at higher levels [61].

The concentrations of heavy metals recorded in this study were much lower than in some reports from China and India, which might be due to lower road traffic, less industrial development and lower population densities in the studied region in comparison to other listed cities. The water used for irrigation of the agricultural land has been shown to affect the levels of heavy metals in the soil and hence crops, as the vegetables grown in the land irrigated by sewage effluents have generally been reported to have higher heavy metal contamination levels. The agricultural land in the Jazan area is mainly irrigated by clean ground water (such as tube-well water), which is considered to be another reason for the lower heavy metal contamination of the vegetables grown in this region. Moreover, the considerably lower Pb concentration observed in this study might be due to the gradual phase-out of leaded petrol in Saudi Arabia. However, this comparison could only be considered approximate because the difference in the sampling procedure and sample digestion methods may influence the results to some extent.

### 3.4. Health Risk Assessment

Low-level intake of heavy metals for a long time has been proved to have a deleterious impact on biological systems, and the harmful effects posed by them become apparent only after several years of exposure [38]. The food chain, through vegetable intake, is considered to be an important exposure pathway of toxic elements to human beings [54]. The vegetables grown in the studied region are mainly sold in the local markets and consumed by local inhabitants. Consequently, the average heavy metal contents recorded in the vegetables were used for the assessment of potential health hazards due to the accumulation of toxic elements through vegetable intake. In this study, the risk assessment was carried out through calculation of the target hazard quotient (THQ).

The estimated daily intakes (EDIs) of toxic metals through the leafy vegetable intake for both adult and child populations were estimated based on average vegetable consumption, and the results are summarized in Table 4. The highest intakes of Cd, Co, Cr, Fe, Mn and Pb were recorded from the leafy vegetables grown around Jazan city, whereas the highest EDIs of Cu and Zn were observed from the Abuarish and Damad regions, respectively. Among the tested heavy metals, the lowest EDIs were recorded for Cd, and the daily intake of Fe was found to be the highest. However, the EDIs of all the tested metals through leafy vegetable intake in this study were less than their respective reference oral doses (RfDs) set by the US EPA, indicating that exposure to these heavy metals through vegetable consumption is unlikely to produce deleterious effects on the consumer population. The calculated THQ values of heavy metals Cd, Co, Cr, Cu, Fe, Mn, Pb and Zn ranged from 9.83 × 10^−2^ to 1.35 × 10^−1^, 2.62 × 10^−3^ to 4.74 × 10^−3^, 1.55 × 10^−1^ to 2.38 × 10^−1^, 3.70 × 10^−2^ to 4.93 × 10^−2^, 1.68 × 10^−1^ to 3.73 × 10^−1^, 3.86 × 10^−1^ to 5.54 × 10^−1^, 6.02 × 10^−2^ to 7.88 × 10^−2^ and 1.90 × 10^−2^ to 2.36 × 10^−2^, respectively, for adults, and 1.40 × 10^−1^ to 1.93 × 10^−1^, 3.74 × 10^−3^ to 6.76 × 10^−3^, 2.22 × 10^−1^ to 3.40 × 10^−1^, 5.29 × 10^−2^ to 7.04 × 10^−2^, 2.40 × 10^−1^ to 5.33 × 10^−1^, 5.50 × 10^−1^ to 7.91 × 10^−1^, 8.58 × 10^−2^ to 1.13 × 10^−1^ and 2.72 × 10^−2^ to 3.36 × 10^−2^, respectively, for children. The THQ values of the heavy metals (for both adults and children) decreased in the order: Mn > Fe > Cr > Cd > Pb > Cu > Zn > Co. Within the different sample collection sites of the studied region, the THQ values were recorded in the following descending order: Jazan > Abuarish > Sabya > Damad > Darb. It has also been noticed that the potential health risks for children were greater than for adults. Overall, the THQ values for all the heavy metals tested in this study were less than 1, which suggested that the vegetables grown in the region are safe to enter into the human food chain and that the consumption of these vegetables posed an insignificant health risk to the local inhabitants. However, in this assessment, only leafy vegetables were considered which only accounts for one type of human food. Other foodstuffs such as other vegetable types, fruits, grains, livestock and seafood might contain considerable amounts of heavy metals which would considerably increase the heavy metal exposure of the local population altogether. Furthermore, many other heavy metal exposure pathways such as the inhalation of urban aerosol, dermal adsorption and the ingestion of contaminated soil (by children while playing) were not included in this study. Mg has not been included in this assessment because it remained below the detection limit in all the tested vegetable samples.

### 3.5. Metal Pollution Index (MPI)

**Figure 4:**
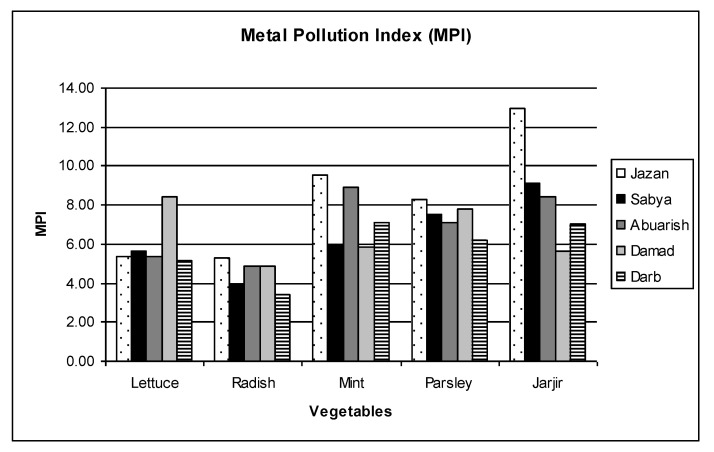
Graph representing the metal pollution index (MPI) of different leafy vegetables in various regions of Jazan province.

The MPI was calculated to estimate the metal pollution load in different vegetables collected from each site, and the results are shown in Figure 4. In general, the MPI values of all vegetables collected from nearby Jazan city were greater than those observed in the samples from other locations, except lettuce collected from the Damad region which showed the highest MPI value. Among the tested vegetables, jarjir had the highest MPI values, followed by mint, parsley and lettuce, while radish exhibited the lowest metal pollution load. It is noteworthy that the MPI values for all the vegetables were different for different regions indicating that the surrounding environment conditions had considerable influence on the concentration of heavy metals in the vegetables. 

### 3.6. Statistical Analysis

A linear regression correlation test was applied to illustrate the relationship between the different heavy metals. The values of the correlation coefficients are depicted in Table 5. Cr showed excellent correlation with Fe and Mn (r = 0.949 and 0.990), and a similar relationship was observed between Mn and Fe (r = 0.978). Cd showed significantly positive correlations with Co, Cr, Fe and Mn with the corresponding r values of 0.568, 0.746, 0.715 and 0.708, respectively. Furthermore, positive relationships were also observed between heavy metals Co-Cr (r = 0.801), Co-Fe (r = 0.774), Co-Mn (r = 0.830), Cu-Pb (r = 0.514) and Cu-Zn (r = 0.792). On the other hand, negative correlations were observed between heavy metals Cd-Cu, Cd-Pb, Cd-Zn, Co-Cu, Co-Zn, Cr-Cu, Cr-Zn, Cu-Fe, Cu-Mn, Fe-Zn and Mn-Zn. Positive correlation between heavy metals suggested that their concentrations in vegetables were probably controlled by similar processes [8]. Such metals probably came into the cultivation land of the studied region and hence the vegetables from two potential sources including atmospheric deposition, fertilizers and insecticides, while the contamination from other heavy metals might be due to human activities or were naturally present in the cultivation soil.

**Figure 5:**
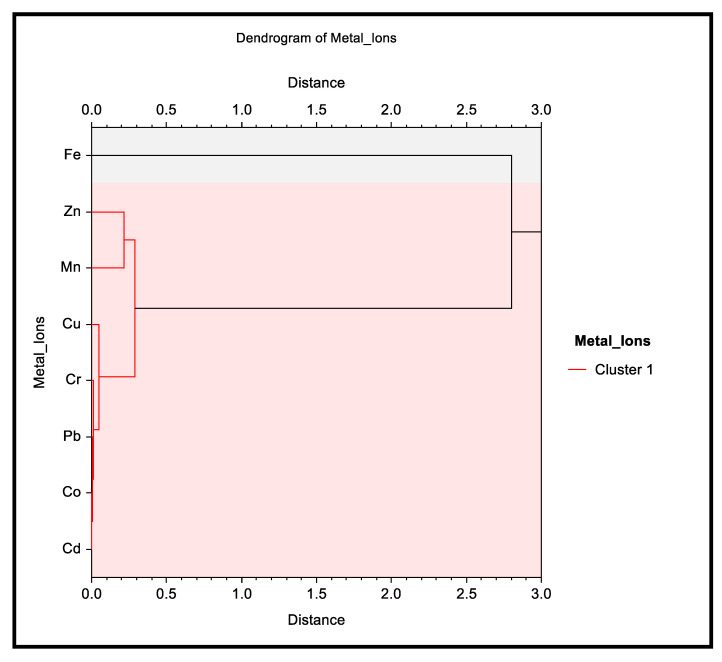
Dendrogram tree obtained after HCA analysis of heavy metals as variables. Only one major cluster was observed showing similar characteristics of the seven tested metals.

The multivariate HCA was performed on the obtained data to identify the variables that have similar characteristics and could have originated from a single source. This method divided the samples on the basis of similarity and placed them into the same cluster. The results obtained are shown as a dendrogram tree in Figure 5. As is evident from the figure, only one cluster was formed consisting of seven out of eight metals. The heavy metals Cd, Co, Cr, Cu, Mn, Pb and Zn are all placed in the same cluster showing similar characteristics among all samples. This showed that all these metals would have originated in different vegetables form a single source. Interestingly, Fe was not placed in any cluster, indicating its dissimilarity and different origin. Further, the similarity between two samples or two groups of samples is denoted by the distance between them: the smaller the distance, the greater the similarity between them. The dendrogram tree showed cluster solutions which were observed as a sudden jump (gap) in the distance coefficients, and the solutions were considered as good solutions if they were present before this gap. The samples separated by a distance value less than 1 were considered to be similar, and as the dissimilarity increased, the distance also increased. Distance values more than 1 indicated that the two variables had different characteristics and could have a different origin. It was clear from the dendrogram tree that Fe was linked to all other metal ions with a distance of 2.80, which showed that it was different from the others. The horizontal lines in the tree signified the distance or dissimilarity between the variables or groups of variables.

Moreover, clustering fit metrics were calculated for the data including the cophenetic correlation and the delta values. These fit metrics indicate the goodness of fit of the data. The cophenetic correlation is one of the Pearson correlation metrics which indicates the correlation between the actual and predicted distances between the tested variables. Generally, a cophenetic correlation value of more than 0.75 is considered appropriate for clustering. The cophenetic correlation value of the presently tested data was found to be 0.9979 which showed excellent correlation between the actual and predicted distance values. The delta values indicate the degree of distortion of the data, and lower delta values closer to zero signify a good fit. There are two delta values calculated: delta (0.5) and delta (1.0), and the configuration with lower delta values fits the data better than the other one. In the present analysis, the two values for delta (0.5) and delta (1.0) were found to be 0.0612 and 0.05240 which showed optimum goodness of fit of the data.

## 4. Conclusions

Leafy vegetables including lettuce, radish, mint, parsley and jarjir grown in the Jazan region of Saudi Arabia were assessed for heavy metal contamination using an ICP-MS. The results revealed that jarjir was the most contaminated, while the lowest heavy metal content was observed in radish. The metal contaminations were found in the following decreasing order: Fe > Mn > Zn > Cu > Cr > Pb > Co > Cd > Mg. Among the selected locations, vegetables collected from cultivation land nearby Jazan city were found to be the most contaminated and those from Darb possessed the lowest levels of heavy metals. Metal pollution index values also suggested that jarjir had the highest pollution level and radish was the least polluted. However, the concentrations of all the tested heavy metals were lower than their corresponding maximum permissible limits set by the FAO/WHO and European Committee. Except Fe and Mn, the levels of all the heavy metals recorded in this study were lower than in other reports from across the globe. Relatively higher Fe and Mn concentrations might be due to their natural abundance in the soil of the region. Based on the EDIs of the heavy metals and the THQ values observed in this study, the vegetables grown in the region are considered to be safe for human intake. The highest and lowest EDIs were recorded for Fe and Cd, respectively, for both adults and children. The EDI values of all the tested heavy metals were lower than their respective reference oral doses (RfDs). The THQ values for both adults and children were calculated to be less than one, suggesting that the local population is unlikely to face obvious health risks due to heavy metal exposure via vegetable consumption. Although the level of heavy metal contamination in the tested vegetables is below the safe limit, long-term exposure of even low doses of toxic elements is a major health concern. This necessitates the elucidation of the mechanistic basis of trace element interactions for health risk assessment. Therefore, further research is required to elucidate the molecular mechanisms and associated human health impact due to exposure to these toxic elements.

## Figures and Tables

**Table 1:** Trace elements and their respective wavelengths.

Elements	Wavelength (nm)
Cd	214.438
Cr	205.560
Co	228.616
Cu	324.754
Mg	279.553
Fe	259.940
Mn	257.610
Pb	220.353
Zn	213.858

**Table 2:** Heavy metal concentrations (mg/kg, on dry weight basis) in leafy vegetables grown in the Jazan region, Saudi Arabia.

Vegetables	Cd	Co	Cr	Cu	Fe	Mg	Mn	Pb	Zn
Jazan (*n* = 4)									
Lettuce	0.24–0.30	0.29–0.46	1.04–2.24	7.95–8.38	239.0–684.5	BDL	36.16–48.02	0.53–0.94	28.78–38.53
Radish	0.22–0.38	0.49–0.74	1.16–1.31	8.11–9.08	222.0–430.7	BDL	32.56–43.00	0.78–1.00	28.03–34.99
Mint	0.18–0.48	0.84–1.12	2.42–4.20	8.05–9.71	995.5–1650.5	BDL	118.70–130.50	1.01–2.09	22.63–35.47
Parsley	0.49–0.70	0.56–0.70	2.78–3.03	6.87–7.54	861.5–1129.0	BDL	76.10–87.60	0.84–2.27	21.93–26.06
Jarjir	1.22–1.49	1.45–1.78	4.46–6.87	5.80–6.29	1996.5–2628.0	BDL	194.80–325.20	0.86–1.16	19.71–20.41
Sabya (*n* = 5)									
Lettuce	0.12–0.40	0.22–0.54	1.57–2.61	5.65–10.11	236.1–760.5	BDL	38.78–54.05	0.55–1.32	26.49–42.11
Radish	0.20–0.40	0.32–0.52	0.48–1.43	2.67–7.54	130.8–188.0	BDL	21.34–30.51	0.49–1.53	16.73–25.19
Mint	0.04–0.20	0.40–0.92	1.69–3.40	5.55–7.34	467.4–1319.0	BDL	69.90–130.75	0.74–1.25	18.80–23.09
Parsley	0.35–0.65	0.34–0.81	1.27–3.65	5.52–12.42	555.5–1366.5	BDL	47.06–98.60	0.68–1.09	22.99–36.01
Jarjir	0.28–1.45	0.48–0.91	1.21–3.81	5.23–7.54	518.0–3400.0	BDL	87.50–158.30	0.89–1.53	12.84–27.82
Abuarish (*n* = 6)									
Lettuce	0.30–0.45	0.35–0.41	0.89–1.07	9.03–10.49	255.4–385.7	BDL	46.19–61.45	0.56–0.85	36.25–45.58
Radish	0.20–0.23	0.37–0.91	0.22–2.33	2.41–7.10	142.9–995.5	BDL	25.96–46.58	0.65–0.80	23.89–26.83
Mint	0.20–0.49	0.85–1.23	2.57–4.69	8.34–12.80	743.5–1091.0	BDL	78.15–118.45	1.01–1.69	25.79–29.78
Parsley	0.18–0.24	0.47–0.63	1.97–2.64	7.00–7.47	1003.0–1250.0	BDL	82.40–103.70	0.71–1.32	27.42–33.13
Jarjir	1.01–2.25	0.40–0.95	1.23–2.37	4.25–13.62	479.3–1106.0	BDL	92.80–153.95	0.73–1.47	11.43–21.54
Damad (*n* = 5)									
Lettuce	0.24–0.43	0.29–1.13	1.29–5.75	9.05–13.20	270.3–987.5	BDL	58.65–60.65	0.82–2.13	31.57–50.20
Radish	0.26–0.36	0.45–0.71	0.45–1.93	1.68–3.38	127.3–810.5	BDL	23.85–39.40	0.89–1.01	25.16–26.20
Mint	0.11–0.33	0.48–0.56	1.09–1.27	7.10–9.00	491.5–610.5	BDL	75.80–106.40	0.75–1.31	24.12–31.27
Parsley	0.22–0.30	0.35–0.83	1.47–4.15	4.81–8.29	551.5–1475.0	BDL	54.30–195.30	0.86–2.03	17.03–36.68
Jarjir	0.66–1.14	0.25–0.66	0.96–1.27	2.95–3.73	294.6–814.0	BDL	42.11–102.30	0.56–0.67	17.74–30.92
Darb (*n* = 6)									
Lettuce	0.22–0.29	0.29–0.50	0.76–0.96	6.98–11.40	253.6–347.6	BDL	54.25–71.45	0.95–1.57	29.31–31.10
Radish	0.16–0.27	0.46–0.47	0.56–1.28	4.14–7.21	33.0–157.5	BDL	21.07–23.98	0.50–0.75	16.29–35.15
Mint	0.17–0.37	0.38–0.84	1.63–6.52	6.87–10.93	496.5–1003.5	BDL	79.00–147.10	0.58–0.90	21.42–30.90
Parsley	0.10–0.45	0.37–0.63	1.26–2.26	4.18–5.65	593.5–977.5	BDL	79.30–96.30	0.78–1.46	19.93–26.71
Jarjir	1.30–1.76	0.35–0.52	1.41–11.91	3.00–3.61	378.8–771.0	BDL	59.65–100.35	0.54–0.70	11.56–17.51

The values represent the average concentration of triplicate runs; *n*: number of plots selected for sample collection; BDL: below detection limit.

**Table 3:** Comparison of analyzed trace elements’ concentrations in vegetables in this investigation with those reported by previous studies.

Unit	Cd	Co	Cr	Cu	Fe	Mg	Mn	Pb	Zn	Location [Ref]
mg/kg, dw	0.10–11.20	N/D	N/D	10.00–111.20	N/D	N/D	N/D	0.50–3.10	29.20–189.10	Varanasi, India [28]
mg/kg, fw	0.003–0.230	N/D	0.078–14.878	N/D	N/D	N/D	N/D	0.003–0.178	N/D	China [30]
mg/kg, dw	0.1–0.6	N/D	N/D	2.5–16.0	N/D	N/D	N/D	1.9–6.6	14.8–49.3	Tanzania [34]
mg/kg, dw	N/D	N/D	N/D	5.42–20.6	166–932	N/D	16.7–42.6	N/D	46.7–91.9	Delhi, India [37]
mg/kg, fw	0.03–0.18	0.07-0.51	BDL-0.48	N/D	N/D	N/D	N/D	0.16–1.66	N/D	Sao Paulo State, Brazil [61]
mg/kg, dw	0.93–4.13	N/D	N/D	2.06–33.22	31.96–543.5	N/D	4.16–94.16	0.54–6.98	8.27–71.77	Saudi Arabia [59]
mg/kg, dw	0.7–2.4	N/D	1.5–6.6	1.0–3.4	N/D	N/D	N/D	0.7–5.4	18–201	Harare, Zimbabwe [60]
mg/kg, fw	0.002–2.918	N/D	N/D	0.155–3.125	N/D	N/D	N/D	0.004–2.361	1.151–54.65	Shizhuyuan, China [38]
mg/kg, dw	0.09–1.55	0.36–1.60	0.86–6.97	2.78–11.58	94.2–2306.2	BDL	22.06–237.72	0.58–1.51	14.79–42.64	Jazan, Saudi Arabia (This study)
mg/kg, fw	0.01–0.13	0.03–0.14	0.07–0.59	0.24–0.99	8.0–196.0	BDL	1.88–20.21	0.05–0.13	1.26–3.63	Jazan, Saudi Arabia (This study)

Conversion factor used to convert the fresh to dry weight of the tested leafy vegetables = 0.085; fw: fresh weight basis; dw: dry weight basis. N/D: not detected; BDL: below detection limit.

**Table 4:** Estimated daily intake (EDI, mg/kg /day), reference dose (RfD, mg/kg /day) and hazard quotient (HQ) of the tested heavy metals in selected leafy vegetables in the Jazan region.

City		Cd	Co	Cr	Cu	Fe	Mn	Pb	Zn
Jazan	EDI (Children)	1.93 × 10^−4^	2.91 × 10^−4^	1.02 × 10^−3^	2.68 × 10^−3^	3.73 × 10^−1^	3.64 × 10^−2^	3.95 × 10^−4^	9.73 × 10^−3^
	HQ (Children)	1.93 × 10^−1^	6.76 × 10^−3^	3.40 × 10^−1^	6.70 × 10^−2^	5.33 × 10^−1^	7.91 × 10^−1^	1.13 × 10^−1^	3.24 × 10^−2^
	EDI (Adult)	1.35 × 10^−4^	2.04 × 10^−4^	7.15 × 10^−4^	1.88 × 10^−3^	2.61 × 10^−1^	2.55 × 10^−2^	2.77 × 10^−4^	6.82 × 10^−3^
	HQ (Adult)	1.35 × 10^−1^	4.74 × 10^−3^	2.38 × 10^−1^	4.69 × 10^−2^	3.73 × 10^−1^	5.54 × 10^−1^	7.91 × 10^−2^	2.27 × 10^−2^
Sabya	EDI (Children)	1.46 × 10^−4^	1.90 × 10^−4^	6.78 × 10^−4^	2.40 × 10^−3^	2.85 × 10^−1^	2.60 × 10^−2^	3.61 × 10^−4^	8.57 × 10^−3^
	HQ (Children)	1.46 × 10^−1^	4.43 × 10^−3^	2.26 × 10^−1^	5.99 × 10^−2^	4.06 × 10^−1^	5.64 × 10^−1^	1.03 × 10^−1^	2.86 × 10^−2^
	EDI (Adult)	1.02 × 10^−4^	1.33 × 10^−4^	4.75 × 10^−4^	1.68 × 10^−3^	1.99 × 10^−1^	1.82 × 10^−2^	2.53 × 10^−4^	6.01 × 10^−3^
	HQ (Adult)	1.02 × 10^−1^	3.10 × 10^−3^	1.58 × 10^−1^	4.20 × 10^−2^	2.85 × 10^−1^	3.95 × 10^−1^	7.22 × 10^−2^	2.00 × 10^−2^
Abuarish	EDI (Children)	1.84 × 10^−4^	2.25 × 10^−4^	6.65 × 10^−4^	2.82 × 10^−3^	2.68 × 10^−1^	2.85 × 10^−2^	3.32 × 10^−4^	9.77 × 10^−3^
	HQ (Children)	1.84 × 10^−1^	5.24 × 10^−3^	2.22 × 10^−1^	7.04 × 10^−2^	3.83 × 10^−1^	6.20 × 10^−1^	9.48 × 10^−2^	3.26 × 10^−2^
	EDI (Adult)	1.29 × 10^−4^	1.58 × 10^−4^	4.66 × 10^−4^	1.97 × 10^−3^	1.88 × 10^−1^	2.00 × 10^−2^	2.32 × 10^−4^	6.84 × 10^−3^
	HQ (Adult)	1.29 × 10^−1^	3.67 × 10^−3^	1.55 × 10^−1^	4.93 × 10^−2^	2.68 × 10^−1^	4.34 × 10^−1^	6.64 × 10^−2^	2.28 × 10^−2^
Damad	EDI (Children)	1.39 × 10^−4^	2.04 × 10^−4^	6.68 × 10^−4^	2.25 × 10^−3^	2.39 × 10^−1^	2.70 × 10^−2^	3.68 × 10^−4^	1.01 × 10^−2^
	HQ (Children)	1.39 × 10^−1^	4.74 × 10^−3^	2.23 × 10^−1^	5.61 × 10^−2^	3.41 × 10^−1^	5.88 ×10^−1^	1.05 × 10^−1^	3.36 × 10^−2^
	EDI (Adult)	9.71 × 10^−5^	1.43 × 10^−4^	4.68 × 10^−4^	1.57 × 10^−3^	1.67 × 10^−1^	1.89 × 10^−2^	2.58 × 10^−4^	7.07 × 10^−3^
	HQ (Adult)	9.71 × 10^−2^	3.32 × 10^−3^	1.56 × 10^−1^	3.93 × 10^−2^	2.39 × 10^−1^	4.12 × 10^−1^	7.37 × 10^−2^	2.36 × 10^−2^
Darb	EDI (Children)	1.81 × 10^−4^	1.61 × 10^−4^	9.79 × 10^−4^	2.11 × 10^−3^	1.68 × 10^−1^	2.53 × 10^−2^	3.00 × 10^−4^	8.14 × 10^−3^
	HQ (Children)	1.81 × 10^−1^	3.74 × 10^−3^	3.26 × 10^−1^	5.29 × 10^−2^	2.40 × 10^−1^	5.50 × 10^−1^	8.59 × 10^−2^	2.71 × 10^−2^
	EDI (Adult)	1.27 × 10^−4^	1.13 × 10^−4^	6.86 × 10^−4^	1.48 × 10^−3^	1.18 × 10^−1^	1.77 × 10^−2^	2.11 × 10^−4^	5.71 × 10^−3^
	HQ (Adult)	1.27 × 10^−1^	2.62 × 10^−3^	2.29 × 10^−1^	3.70 × 10^−2^	1.68 × 10^−1^	3.86 × 10^−1^	6.02 × 10^−2^	1.90 × 10^−2^
RfD		1.00 × 10^−3^	4.30 × 10^−2^	3.00 × 10^−3^	4.00 × 10^−2^	7.00 × 10^−1^	4.60 × 10^−2^	3.50 × 10^−3^	3.00 × 10^−1^

The health risk due to Mg was not estimated because it remained undetected in all the leafy vegetable samples.

**Table 5:** Correlations between heavy metal concentrations of tested leafy vegetables.

	Cd	Co	Cr	Cu	Fe	Mn	Pb	Zn
Cd	1.000							
Co	0.568	1.000						
Cr	0.746	0.801	1.000					
Cu	−0.538	−0.326	−0.063	1.000				
Fe	0.715	0.774	0.949	−0.215	1.000			
Mn	0.708	0.830	0.990	−0.111	0.978	1.000		
Pb	−0.285	0.128	0.321	0.514	0.430	0.392	1.000	
Zn	−0.716	−0.819	−0.601	0.792	−0.700	−0.654	0.131	1.000

Number of vegetables included in this test = 5. Number of values for correlation = 10.

## Data Availability

The data presented in this study are available on request from the corresponding author.
